# Comparison of levetiracetam with phenytoin for the prevention of intravenous busulfan-induced seizures in hematopoietic cell transplantation recipients

**DOI:** 10.1007/s00280-018-3659-8

**Published:** 2018-08-06

**Authors:** Kana Akiyama, Tetsuo Kume, Masafumi Fukaya, Ikue Shiki, Terukazu Enami, Raine Tatara, Michihiro Shino, Takashi Ikeda

**Affiliations:** 1 Department of Pharmacy, Shizuoka Cancer Center, 1007, Shimonagakubo, Nagaizumi-cho, Sunto-gun, Shizuoka, 411-8777 Japan; 2 Division of Hematology and Stem Cell Transplantation, Shizuoka Cancer Center, 1007, Shimonagakubo, Nagaizumi-cho, Sunto-gun, Shizuoka, 411-8777 Japan

**Keywords:** Anticonvulsant, Intravenous busulfan, Seizures, Stem cell transplantation

## Abstract

**Purpose:**

Busulfan is used as a conditioning regimen for hematopoietic stem cell transplantation and is known to cause seizures as a side effect. As various anticonvulsant drugs have been reported, we conducted a retrospective investigation regarding the preventive effects and adverse events associated with different anticonvulsants administered alongside intravenous busulfan (ivBu) in our institution.

**Methods:**

We targeted 104 patients who received ivBu at our institution from May 1, 2010 to April 30, 2017. We investigated the seizure prevention rate and adverse events rate under anticonvulsant prophylaxis.

**Results:**

There were 70 cases (67.3%) of phenytoin administration and 34 cases (32.7%) of levetiracetam administration for anticonvulsant therapy. The seizure prevention rate was 98.6% for phenytoin and 100% for levetiracetam; seizures occurred in one out of 104 patients. There were no significant differences in the seizure prevention rate depending on the type of anticonvulsant. Further, there were no differences in adverse events.

**Conclusions:**

Anticonvulsant prophylaxis is considered necessary for safe conditioning with ivBu. Adverse events associated with the use of levetiracetam are within an acceptable range. Further, levetiracetam is considered useful as a preventive drug against seizures during ivBu administration because it is easy to administer and has ideal pharmacokinetics for supportive care.

## Introduction

Intravenous busulfan (ivBu) has been used as a conditioning regimen for hematopoietic stem cell transplantation [1–3]. Busulfan is known to cause seizures; at high doses it passes freely through the blood–brain barrier, resulting in high concentrations in the central nervous system, thus increasing the risk of convulsive seizures. Busulfan-induced seizures are generally tonic-clonic in nature. It has been reported that the incidence of seizures is 10% (range 1.8–40%) when high-dose busulfan is used without preventive measures [4]. Drugs used to prevent busulfan-induced seizures must be fast acting, should not increase the toxicity of the conditioning agents, and should not alter the pharmacokinetics of the conditioning regimen [4, 5]. While the package insert for busulfan recommends anticonvulsant prophylaxis, no information regarding a specific drug or its administration is provided. There are many reports describing the use of antiepileptic drugs, such as phenytoin and valproic acid, as well as benzodiazepines, such as clonazepam or lorazepam, as prophylactic treatments against busulfan-induced seizures [4, 6, 7]. While there are many reported cases of phenytoin use, it reportedly induces drug metabolizing enzymes, thereby interacting with the metabolism of cyclophosphamide, a drug used in the conditioning regimen [8, 9]. More recently, there have been reports of the use of levetiracetam as a prophylaxis against busulfan-induced seizures in pediatrics; however, there is little information regarding its use in adults [5, 10, 11]. The pharmacokinetic features of levetiracetam, including approximately 100% bioavailability and linear pharmacokinetics, enable its usage without therapeutic drug monitoring (TDM) [12, 13]. Additionally, the drug has no effect on cytochrome P450 metabolic enzymes [14]. In this study, we conducted a retrospective analysis of prophylactic anticonvulsant agents, as well as their adverse effects, when used in cases of ivBu administration at our institution.

## Materials and methods

We included 104 cases of ivBu usage during hematopoietic stem cell transplantation at the Shizuoka Cancer Center from May 1, 2010 to April 30, 2017 in this study. The main study endpoints were seizure and involuntary movement prevention rates during ivBu administration. In addition, the presence of nausea, vomiting, and oral mucositis was assessed from the start of the conditioning regimen to the day of transplantation. Oral mucositis, total bilirubin, and veno-occlusive disease/sinusoidal obstruction syndrome (VOD/SOS) were assessed from the start of the conditioning regimen to 28 days post-transplantation as secondary endpoints to confirm that ivBu-induced adverse events were not enhanced by the use of anticonvulsants. Moreover, neutrophil engraftment was assessed. Neutrophil engraftment was defined as the first day of an absolute neutrophil count > 500/µL on three consecutive measurements. The Common Terminology Criteria for Adverse Events (version 4.0) and the Modified Seattle Criteria were used for retrospective assessment of the medical records [15, 16].

For this type of study formal consent is not required.

### Statistical analysis

We used Fisher’s exact test, and *p* values < 0.05 were considered statistically significant. All statistical analyses were performed with EZR version 3.2.2; EZR is a modified version of “R Commander” that includes statistical functions frequently used in biostatistics.

## Results

The patient characteristics are shown in Table 1. A diagnosis of “other” included anaplastic large cell lymphoma, mixed phenotype acute leukemia B/myeloid, Hodgkin’s lymphoma/nodular sclerosis, myelofibrosis, and adult T-cell leukemia/lymphoma, representative of one individual each. The ivBu2 + α conditioning regimen included the use of intravenous busulfan at 6.4 mg/kg in combination with another drug. For the ivBu4 + β conditioning regimen, ivBu was administered at 12.8 mg/kg in combination with another drug.

**Table 1:** Patient characteristics as *N* (%) or median (range)

	PHT	LEV
*N*	70	34
Male sex	46 (65.7)	18 (52.9)
Age (years)	58 (17–74)	58 (35–69)
Diagnosis		
AML	40 (57.1)	10 (29.4)
MDS	24 (34.3)	11 (32.4)
CML	4 (5.7)	3 (8.8)
PCNSL	0	5 (14.7)
DLBCL	2 (2.9)	0
Other	0	5 (14.7)
Donor/stem cell source		
Autologous peripheral blood	0	5 (14.7)
Related peripheral blood	14 (20.0)	4 (11.8)
Unrelated peripheral blood	0	5 (14.7)
Unrelated bone marrow	42 (60.0)	10 (29.4)
Unrelated cord blood	14 (20.0)	10 (29.4)
Conditioning regimen		
ivBu/CY	20 (28.6)	9 (26.5)
FB4	8 (11.4)	0
ATG/FB4 ± AraC	37 (52.9)	18 (52.9)
FB4/TBI ± AraC	2 (2.9)	4 (11.8)
ivBu2 + α	3 (4.3)	0
ivBu4 + β	0	3 (8.8)
Emesis prophylaxis		
Granisetron	70 (100)	10 (29.4)
Palonosetron	0	1 (2.9)
Palonosetron + fosaprepitant	0	23 (67.6)

*AML* acute myeloid leukemia,* MDS* myelodysplastic syndrome,* CML* chronic myeloid leukemia, *PCNSL* primary central nervous system lymphoma,* DLBCL* diffuse large B cell lymphoma,* ivBu/CY* intravenous busulfan at 12.8 mg/kg + cyclophosphamide,* ivBu2 + α* intravenous busulfan at 6.4 mg/kg + other drugs,* FB4* fludarabine + intravenous busulfan at 12.8 mg/kg,* ATG* anti-thymocyte globulin (rabbit),* AraC* cytarabine,* TBI* total body irradiation,* ivBu4 + β* intravenous busulfan at 12.8 mg/kg + other drugs,* PHT* phenytoin,* LEV* levetiracetam.

Phenytoin and levetiracetam were used as anticonvulsants in 70 (67.3%) and 34 (32.7%) cases, respectively. All cases of prophylaxis using phenytoin were administered intravenously. One case of prophylaxis using phenytoin was started on the initial day of ivBu administration, while all other cases were started the day before ivBu administration. The dose of phenytoin before ivBu administration was 125 mg/day in one case, 150 mg/day in two cases, and 300 mg/day in the remaining cases. After initiation of ivBu administration, the dose of phenytoin was 250 mg/day in two cases and 300 mg/day in the remaining cases. All cases of prophylaxis using oral levetiracetam were given 1000 mg/day and began 2 days prior to the start of ivBu administration. Phenytoin or levetiracetam was administered as a prophylactic treatment until the day after the termination of ivBu administration.

Under phenytoin anticonvulsant prophylaxis, the seizure and involuntary movement prevention rates were 98.6 and 87.1%, respectively. Prevention rates associated with levetiracetam were 100 and 97.1% for seizures and involuntary movements, respectively. There was one confirmed case of a seizure from a patient having received 125 mg/day phenytoin 1 day before ivBu administration; the seizure was identified on the second day of ivBu administration, at which point the conditioning regimen was changed by the attending physician in consideration of the difficulties associated with continuing the existing regimen.

Assessments of the incidence of nausea, vomiting, and oral mucositis are shown in Table 2. Grade one (Gr1) oral mucositis was found in 55.7% and Gr2 oral mucositis was found in 2.9% of patients receiving phenytoin prophylaxis, from the start of the conditioning regimen to transplantation day; in contrast, Gr1 oral mucositis was found in 38.2% and Gr2 oral mucositis was found in 0% of patients receiving levetiracetam prophylaxis (*p* = 0.12). Gr3 oral mucositis was not observed in either prophylactic treatment group. However, the incidences of oral mucositis in the phenytoin prophylaxis groups were Gr1: 31.4%, Gr2: 25.7%, and Gr3: ≤ 35.7% from the start of the conditioning period to 28 days post-transplantation; incidences of oral mucositis were Gr1: 14.7%, Gr2: 44.1%, and Gr3: ≤ 35.3% (*p* = 0.18) in the levetiracetam prophylaxis group. There were no significant differences in the occurrence of adverse events, including nausea, vomiting, oral mucositis, and liver dysfunction based on differences in anticonvulsant usage.

**Table 2:** Incidence of nausea, vomiting, oral mucositis, and liver toxicity, *N* (%)

	PHT (*n* = 70)	LEV (*n* = 34)	*p* value
Nausea			
All grades	50 (71.4)	30 (88.2)	0.08
Grade 3 ≤	12 (17.1)	1 (2.9)	0.06
Vomiting			
All grades	34 (48.6)	18 (52.9)	0.84
Grade 3 ≤	3 (4.3)	0	0.55
Mucositis oral			
Until transplant day			
All grades	41 (58.6)	13 (38.2)	0.06
Grade 3 ≤	0	0	
Until day 28			
All grades	65 (92.9)	32 (94.1)	1
Grade 3 ≤	25 (35.7)	12 (35.3)	1
T-Bil			
All grades	29 (41.4)	10 (29.4)	0.28
Grade 3 ≤	2 (2.9)	0	1
VOD/SOS	2 (2.9)	0	1

*PHT* phenytoin, *LEV* levetiracetam, *T-Bil* total bilirubin, *VOD/SOS* veno-occlusive disease/sinusoidal obstruction syndrome

The median number of days to neutrophil engraftment was 15.5 (range 11–24) days and 15.0 (10–31) days in phenytoin and levetiracetam groups, respectively.

## Discussion

We conducted a retrospective study to assess the types of anticonvulsant drugs and their prophylactic effects when used in cases of ivBu administration. Previous studies have reported that seizures occur at a frequency of 1–40% in cases of busulfan administration with no prophylactic anticonvulsant use [4, 17]. In our study, seizure prevention rates of 98.6 and 100% were observed when phenytoin and levetiracetam were used as prophylactic treatments against ivBu-induced seizures, respectively. There were no statistically significant differences between anticonvulsant types. However, a confirmed seizure case was observed with phenytoin prophylaxis, resulting in difficulty with the use of ivBu and a subsequent change in conditioning agent. Previous reports have identified a 1% frequency in the incidence of seizures even under prophylactic anticonvulsant administration [18, 19]. ivBu-induced seizures may occur even in the presence of anticonvulsant agents, resulting in a sudden change in the conditioning schedule. Therefore, there is a need for additional prophylactic anticonvulsants to prevent ivBu-induced seizures and avoid sudden changes in the conditioning schedule.

To be used as a prophylactic agent, a drug should not enhance the toxicity associated with the conditioning regimen [4]. No statistically significant differences in nausea and vomiting were observed between the anticonvulsants studied, during the conditioning regimen. The incidence of vomiting in the levetiracetam prophylaxis group was less than that of previous reports [1, 20]. The combination of palonosetron + fosaprepitant was used for its antiemetic effect in 67.6% of patients in the levetiracetam prophylaxis group. Palonosetron is a second-generation 5-hydroxytryptamine 3 receptor antagonist that is more effective in the suppression of delayed nausea and vomiting than the first-generation 5-hydroxytryptamine 3 receptor antagonist, granisetron [21]. In addition, nausea and vomiting are alleviated by a combination of 5-hydroxytryptamine 3 receptor antagonists and the neurokinin-1 receptor antagonist, fosaprepitant; fosaprepitant is the pro-drug of aprepitant [22, 23]. The ability to use both substances in recent years has resulted in improvements in the control of vomiting over that observed in past reports. This is in contrast to oral mucositis, with no noted differences in its high incidence rate. Moreover, the presentation of VOD/SOS did not differ significantly from previous reports [6, 10, 20, 24]. Furthermore, the median number of days to neutrophil engraftment did not differ significantly in a previous systematic review [25], and it may be assumed that anticonvulsant administration had no significant effects on adverse events associated with ivBu administration.

This study assessed the use of phenytoin or levetiracetam as anticonvulsant agents. TDM is sometimes required with phenytoin, as it displays non-linear pharmacokinetics. In contrast, levetiracetam is approximately 100% bioavailable, with no effects on cytochrome P450 metabolic enzymes. Consequently, levetiracetam can be used orally or intravenously without consideration of the cytochrome P450 enzyme system or TDM [12–14]. While slight involuntary movements were observed, there was an absence of seizures in the levetiracetam prophylaxis group. An oral mucositis score of Gr2 or less was observed from the start of conditioning to transplantation, enabling drugs to be administered orally throughout the ivBu administration period. Oral levetiracetam can be administered continually as a prophylactic agent against ivBu-induced seizures; therefore, medical personnel may consider oral levetiracetam administration to be a more convenient method than that required for phenytoin.

Limitations exist in the current study. First, this was a retrospective study carried out in a single institution with a limit to the drugs that could be used as anticonvulsants. The second was the inability to conduct pharmacokinetic monitoring of ivBu. As such, we were unable to evaluate the effects of phenytoin and levetiracetam on ivBu pharmacodynamics. The third limitation concerns difficulties in adjusting the dose of phenytoin, attributable to the short administration period, with a consequent inability to conduct TDM for phenytoin. Therefore, it is unclear whether optimal blood concentrations of phenytoin were maintained during ivBu administration. Fourth, direct hematological toxicity induced by levetiracetam could not be evaluated owing to strong myelosuppression of myeloablative conditioning [26, 27].

In conclusion, prophylactic anticonvulsant administration is necessary to facilitate transplantations without sudden changes in conditioning schedules. Further, our results show that levetiracetam can be used to effectively prevent busulfan-induced seizures in adults. Moreover, the use of levetiracetam is convenient for medical staff, as it can be administered orally and TDM is not required. Importantly, the absence of adverse events induced with oral administration of levetiracetam during ivBu administration is indicative of its application for continued administration and its convenience of use by medical staff. These results support clinical decision making by providing evidence for the use of levetiracetam during ivBu conditioning. In the future, it is necessary to consider prospective studies to validate supportive care for better busulfan-induced seizure prevention.

## References

[1] Kim SW, Mori SI, Tanosaki R, Fukuda T, Kami M, Sakamaki H, Yamashita T, Kodera Y, Terakura S, Taniguchi S, Miyakoshi S, Usui N, Yano S, Kawano Y, Nagatoshi Y, Harada M, Morishima Y, Okamoto S, Saito AM, Ohashi Y, Ueda R, Takaue Y (2009). Busulfex (i.v. BU) and CY regimen before SCT: Japanese-targeted phase II pharmacokinetics combined study. Bone Marrow Transplant.

[2] Bredeson C, LeRademacher J, Kato K, Dipersio JF, Agura E, Devine SM, Appelbaum FR, Tomblyn MR, Laport GG, Zhu X, McCarthy PL, Ho VT, Cooke KR, Armstrong E, Smith A, Rizzo JD, Burkart JM, Pasquini MC (2013). Prospective cohort study comparing intravenous busulfan to total body irradiation in hematopoietic cell transplantation. Blood.

[3] Lee JH, Joo YD, Kim H, Ryoo HM, Kim MK, Lee GW, Lee JH, Lee WS, Park JH, Bae SH, Hyun MS, Kim DY, Kim SD, Min YJ, Lee KH (2013). Randomized trial of myeloablative conditioning regimens: busulfan plus cyclophosphamide versus busulfan plus fludarabine. J Clin Oncol.

[4] Eberly AL, Anderson GD, Bubalo JS, McCune JS (2008). Optimal prevention of seizures induced by high-dose busulfan. Pharmacotherapy.

[5] Floeter AE, McCune JS (2017). Levetiracetam for the prevention of busulfan-induced seizures in pediatric hematopoietic cell transplantation recipients. J Oncol Pharm Pract.

[6] Sato M, Kako S, Matsumoto K, Oshima K, Akahoshi Y, Nakano H, Ugai T, Yamasaki R, Wada H, Ishihara Y, Sakamoto K, Kawamura K, Ashizawa M, Terasako-Saito K, Kimura S, Nakasone H, Kikuchi M, Tanihara A, Yamazaki R, Tanaka Y, Kanda J, Nishida J, Morita K, Kanda Y (2015). Pharmacokinetics study of once-daily intravenous busulfan in conditioning regimens for hematopoietic stem cell transplantation. Int J Hematol.

[7] Diaz-Carrasco MS, Olmos R, Blanquer M, Velasco J, Sánchez-Salinas A, Moraleda JM (2013). Clonazepam for seizure prophylaxis in adult patients treated with high dose busulfan. Int J Clin Pharm.

[8] Slattery JT, Kalhorn TF, McDonald GB, Lambert K, Buckner CD, Bensinger WI, Anasetti C, Appelbaum FR (1996). Conditioning regimen-dependent disposition of cyclophosphamide and hydroxycyclophosphamide in human marrow transplantation patients. J Clin Oncol.

[9] de Jonge ME, Huitema AD, van Dam SM, Beijnen JH, Rodenhuis S (2005). Significant induction of cyclophosphamide and thiotepa metabolism by phenytoin. Cancer Chemother Pharmacol.

[10] Soni S, Skeens M, Termuhlen AM, Bajwa RP, Gross TG, Pai V (2012). Levetiracetam for busulfan-induced seizure prophylaxis in children undergoing hematopoietic stem cell transplantation. Pediatr Blood Cancer.

[11] Yazal Erdem A, Azık F, Tavil B, Teber S, Tunç B, Uçkan D (2014). Busulfan triggers epileptic seizures under levetiracetam and valproic acid therapy. Pediatr Transplant.

[12] Ramael S, De Smedt F, Toublanc N, Otoul C, Boulanger P, Riethuisen JM, Stockis A (2006). Single-dose bioavailability of levetiracetam intravenous infusion relative to oral tablets and multiple-dose pharmacokinetics and tolerability of levetiracetam intravenous infusion compared with placebo in healthy subjects. Clin Ther.

[13] Tan J, Paquette V, Levine M, Ensom MHH (2017). Levetiracetam clinical pharmacokinetic monitoring in pediatric patients with epilepsy. Clin Pharmacokinet.

[14] Perucca E, Gidal BE, Baltès E (2003). Effects of antiepileptic comedication on levetiracetam pharmacokinetics: a pooled analysis of data from randomized adjunctive therapy trials. Epilepsy Res.

[15] Common Terminology Criteria for Adverse Events, version 4.0. NCI, NIH, DHHS. https://evs.nci.nih.gov/ftp1/CTCAE/CTCAE_4.03/CTCAE_4.03_2010-06-14_QuickReference_5x7.pdf. Accessed 8 May 2017

[16] McDonald GB, Hinds MS, Fisher LD, Schoch HG, Wolford JL, Banaji M, Hardin BJ, Shulman HM, Clift RA (1993). Veno-occlusive disease of the liver and multiorgan failure after bone marrow transplantation: a cohort study of 355 patients. Ann Intern Med.

[17] Leon-Rodriguez E, Rivera-Franco MM (2016). Minimal incidence of neurotoxicity without prophylaxis during busulfan-based conditioning regimen in patients undergoing stem cell transplantation. Int J Hematol.

[18] De La Camara R, Tomas JF, Figuera A, Berberana M, Fernandez-Rañada JM (1991). High dose busulfan and seizures. Bone Marrow Transplant.

[19] Caselli D, Rosati A, Faraci M, Podda M, Ripaldi M, Longoni D, Cesaro S, Lo Nigro L, Paolicchi O, Maximova N, Menconi MC, Ziino O, Cicalese MP, Santarone S, Nesi F, Aricò M, Locatelli F, Prete A (2014). Risk of seizures in children receiving busulphan-containing regimens for stem cell transplantation. Biol Blood Marrow Transplant.

[20] Andersson BS, Kashyap A, Gian V, Wingard JR, Fernandez H, Cagnoni PJ, Jones RB, Tarantolo S, Hu WW, Blume KG, Forman SJ, Champlin RE (2002). Conditioning therapy with intravenous busulfan and cyclophosphamide (IV BuCy2) for hematologic malignancies prior to allogeneic stem cell transplantation: a phase II study. Biol Blood Marrow Transplant.

[21] Saito M, Aogi K, Sekine I, Yoshizawa H, Yanagita Y, Sakai H, Inoue K, Kitagawa C, Ogura T, Mitsuhashi S (2009). Palonosetron plus dexamethasone versus granisetron plus dexamethasone for prevention of nausea and vomiting during chemotherapy: a double-blind, double-dummy, randomised, comparative phase III trial. Lancet oncol.

[22] Uchida M, Kato K, Ikesue H, Ichinose K, Hiraiwa H, Sakurai A, Muta T, Takenaka K, Iwasaki H, Miyamoto T, Teshima T, Shiratsuchi M, Suetsugu K, Nagata K, Egashira N, Akashi K, Oishi R (2013). Efficacy and safety of aprepitant in allogeneic hematopoietic stem cell transplantation. Pharmacotherapy.

[23] Uchida M, Ikesue H, Miyamoto T, Kato K, Suetsugu K, Ichinose K, Hiraiwa H, Sakurai A, Takenaka K, Muta T, Iwasaki H, Teshima T, Shiratsuchi M, Egashira N, Akashi K, Oishi R (2013). Effectiveness and safety of antiemetic aprepitant in Japanese patients receiving high-dose chemotherapy prior to autologous hematopoietic stem cell transplantation. Biol Pharm Bull.

[24] Chaudhry H, Bruce AJ, Wolf RC, Litzow MR, Hogan WJ, Patnaik MS, Kremers WK, Phillips GL, Hashmi SK (2016). The incidence and severity of oral mucositis among allogeneic hematopoietic stem cell transplantation patients: a systematic review. Biol Blood Marrow Transplant.

[25] Ben Barouch S, Cohen O, Vidal L, Avivi I, Ram R (2016). Busulfan fludarabine vs busulfan cyclophosphamide as a preparative regimen before allogeneic hematopoietic cell transplantation: systematic review and meta-analysis. Bone Marrow Transplant.

[26] Bacigalupo A, Ballen K, Rizzo D, Giralt S, Lazarus H, Ho V, Apperley J, Slavin S, Pasquini M, Sandmaier BM, Barrett J, Blaise D, Lowski R, Horowitz M (2009). Defining the intensity of conditioning regimens: working definitions. Biol Blood Marrow Transplant.

[27] Léger F, Nguyen L, Puozzo C (2009). Exposure equivalence between IV (0.8 mg/kg) and oral (1 mg/kg) busulfan in adult patients. Eur J Clin Pharmacol.

